# First-Principles Investigation of Four-Phonon Scattering Effects on Thermal Transport in Two-Dimensional BeN_4_

**DOI:** 10.3390/ma19122572

**Published:** 2026-06-14

**Authors:** Ziqing Ji, Lei Hao, Weiqi Cai, Xinyu Wang, Ziman Wang

**Affiliations:** 1 Institute of Thermal Science and Technology, Shandong University, Jinan 250061, China; 2 School of Electronic and Information Engineering, Beihang University, Beijing 100191, China; 3 Key Laboratory of Material Physics and Chemistry of the Ministry of Education Under Extraordinary Conditions, School of Physical Science and Technology, Northwestern Polytechnical University, Xi’an 710129, China; 4 Research Institute for Special Structures of Aeronautical Composites, Aviation Industry Corporation of China, Ltd., Jinan 250023, China; 5 Shandong Province Key Laboratory for Electromagnetic Control and Multifunctional Integration Technology of Aerospace Electromagnetic Functional Structure, Jinan 250023, China; 6 Shenzhen Research Institute of Shandong University, Shenzhen 518057, China

**Keywords:** two-dimensional material, lattice thermal conductivity, four-phonon scattering, first-principles calculation

## Abstract

Four-phonon (4 ph) scattering is critically important for describing thermal transport properties in two-dimensional (2D) materials. Incorporating the 4 ph process is crucial for obtaining reliable lattice thermal conductivity (*κ*_l_) and understanding phonon thermal transport. Among emerging 2D materials, monolayer BeN_4_ has attracted increasing attention because of its unique structural properties. Here, the influence of 4 ph scattering on the thermal transport behavior of monolayer BeN_4_ is comprehensively explored through first-principles calculations. The calculated results demonstrate that, after considering the 4 ph scattering, the *κ*_l_ of monolayer BeN_4_ at 300 K are reduced by 37.7% and 50.6% along the zigzag and armchair directions, respectively. These findings indicate that monolayer BeN_4_ exhibits anisotropy in thermal transport and that 4 ph scattering has a significant impact on thermal transport. The thermal transport is dominated by acoustic phonon branches. Furthermore, the larger *κ*_l_ at low temperatures originates from longer phonon lifetimes, larger phonon mean free paths, lower phonon scattering rates, and smaller weighted phase space. In addition, the different channels of 4 ph scattering are systematically analyzed, revealing that the redistribution channel provides the dominant contribution to 4 ph scattering. This investigation provides deeper insight into the thermal transport behavior of monolayer BeN_4_ and facilitates its potential applications in nanoelectronic and thermal management devices.

## 1. Introduction

Since Geim and Novoselov successfully isolated graphene in 2004, two-dimensional (2D) materials have become an important research focus owing to their remarkable physical and chemical characteristics [1,2,3]. 2D materials exhibit remarkable characteristics, including excellent electronic performance [4,5], outstanding mechanical flexibility [6,7], high optical transparency [8,9], and facile fabrication [10,11]. Benefiting from these advantages, 2D materials have shown broad application prospects in electronic devices, photonic devices, energy conversion devices, and nanoscale thermal management systems [12,13,14,15,16]. In recent years, extensive efforts have been devoted to exploring novel 2D materials with diverse crystal structures and electronic properties to further expand their applications in next-generation nanoelectronic technologies [17,18]. Meanwhile, understanding lattice thermal transport in low-dimensional materials is essential for improving the performance and reliability of nanoscale devices.

Monolayer BeN_4_ has been successfully synthesized under high-pressure conditions and subsequently stabilized upon release to ambient pressure [19]. As a nitrogen-rich 2D Dirac material, BeN_4_ has attracted considerable attention because of its unique crystal structure and intriguing electronic properties [20,21]. The BeN_4_ monolayer exhibits intrinsically anisotropic massless Dirac fermions and Fermi velocities, with P2/m symmetry [19]. In addition to its electronic structure and mechanical stability [22,23], the thermal transport properties of BeN_4_ have also been investigated. Previous studies have demonstrated that monolayer BeN_4_ possesses relatively high lattice thermal conductivity, suggesting its potential applications in nanoelectronic thermal management [22,23,24,25]. However, despite the progress achieved in understanding the thermal transport properties of BeN_4_, the effects of four-phonon (4 ph) scattering on its lattice thermal conductivity have not yet been systematically explored.

Previous investigations have shown that higher-order anharmonic scattering, particularly 4 ph scattering, can substantially influence thermal transport in 2D materials [26,27]. For example, after considering 4 ph scattering, the predicted lattice thermal conductivity of graphene decreases dramatically from 3544 to 810 W m^−1^ K^−1^ [28], while the corresponding value for Bas [29] is reduced from 2200 to 1400 W m^−1^ K^−1^. These results indicate that 4 ph scattering has a non-negligible influence on thermal transport in materials. Furthermore, for some materials, elevated temperature can significantly enhance 4 ph scattering effects [29,30,31]. Considering only three-phonon (3 ph) scattering is insufficient to accurately describe thermal transport behavior, making it essential to investigate the temperature dependence of thermal transport. For instance, 4 ph scattering can reduce the predicted thermal conductivity of Si by 25% at 1000 K but only 8% at 300 K [29]. This discrepancy suggests that thermal transport mechanisms are more complex at elevated temperatures and require a more comprehensive description. Therefore, a comprehensive investigation of the thermal transport mechanism in 2D BeN_4_, with both 3 ph and 4 ph scattering taken into account, is necessary.

In this work, we systematically investigate the effects of 4 ph scattering on the lattice thermal conductivity (*κ*_l_) of monolayer BeN_4_ based on first-principles calculations and the Boltzmann transport equation. The optimized crystal structure and calculated phonon dispersion are obtained to verify the dynamical stability of monolayer BeN_4_. Subsequently, the *κ*_l_ considering only 3 ph scattering is evaluated, followed by a further analysis incorporating the contribution of the 4 ph scattering process. Furthermore, to reveal the microscopic origin of thermal transport, the phonon behaviors are investigated, which consist of the phonon group velocity, phonon lifetime, phonon mean free path, phonon scattering rate, and weighted phase space. Meanwhile, the distinct processes of 3 ph and 4 ph scattering are examined. These include the emission and absorption processes in 3 ph scattering, as well as the redistribution, splitting, and recombination processes in 4 ph scattering. Finally, the Grüneisen parameter is evaluated to assess the influence of temperature on thermal transport. This study elucidates the influence of 4 ph scattering on *κ*_l_ of 2D BeN_4_, and offers deeper insight into the underlying phonon scattering behavior.

## 2. Methods

All calculations are conducted within the framework of density functional theory (DFT) employing the Vienna Ab initio Simulation Package (VASP 5.4.4) [32]. The exchange-correlation interaction is described by the generalized gradient approximation (GGA) in the Perdew−Burke−Ernzerhof (PBE) function for monolayer BeN_4_. A kinetic energy cutoff of 500 eV is adopted for the plane-wave basis set. Structural optimization is performed until the residual atomic forces and total energy variations are below 10^−3^ eV Å^−1^ and 10^−6^ eV, respectively. A 10 × 10 × 1 k-mesh is used for Brillouin zone sampling. To suppress interactions between neighboring periodic layers, a vacuum region of 20 Å is set along the perpendicular direction (*z*) of the monolayer. The second-order interatomic force constants (IFCs) are generated from a 4 × 4 × 1 supercell with the PHONOPY package [33]. The anharmonic third-order and fourth-order IFCs are extracted using 3 × 3 × 1 supercells with the Third-order [34] and Fourth-order [35] scripts, respectively.

The *κ*_l_ considering 3 ph and 4 ph scattering contributions is obtained through iterative calculations of the phonon Boltzmann transport equation (BTE) implemented in the ShengBTE v1.2.0 [34] and FourPhonon [35] packages, respectively. The calculations are performed on 70 × 70 × 1 and 20 × 20 × 1 q-meshes for the 3 ph and 4 ph processes, respectively. The consistency with literature convergence studies and the good agreement with previous theoretical data support that the chosen mesh is adequate for the present analysis [36,37,38]. The BTE equation is shown as

(1) $$\kappa_{l}=\kappa_{\alpha \alpha }=\frac{1}{NV}\sum_{\lambda }\frac{\partial f_{\lambda }}{\partial T}\left(\hbar \omega_{\lambda }\right)v_{\lambda }^{2}\tau_{\lambda \alpha },$$

where *V*, *f_λ_*, $v_{\lambda }$ and *τ_λα_* are the volume of the unit cell, the Bose–Einstein distribution function, the harmonic group velocity, and the anharmonic relaxation time, respectively. The reciprocal of the total phonon lifetime corresponds to the total phonon scattering rate, which includes contributions from all scattering channels according to Matthiessen’s rule:

(2) $$\frac{1}{\tau_{\lambda }}=\frac{1}{\tau_{\lambda }^{3ph}}+\frac{1}{\tau_{\lambda }^{4ph}},$$

where $\frac{1}{\tau_{\lambda }^{3ph}}$ and $\frac{1}{\tau_{\lambda }^{4ph}}$ represents the three-phonon scattering rate and four-phonon scattering rate, respectively. The $\frac{1}{\tau_{\lambda }^{3ph}}$ can be expressed as the summation of the three-phonon transition probabilities $\Gamma_{\lambda \lambda^{\prime }\lambda^{\prime \prime }}^{\pm }$, which can be evaluated according to the following equation [34]:


(3) $$\Gamma_{\lambda \lambda^{\prime }\lambda^{\prime \prime }}^{\pm }=\frac{\hbar \pi }{4}\left\{\begin{array}{l} f_{\lambda^{\prime }}-f_{\lambda^{\prime \prime }}\\ f_{\lambda^{\prime }}+f_{\lambda^{\prime \prime }}+1 \end{array}\right\}\frac{\delta \left(\omega_{\lambda }\pm \omega_{\lambda^{\prime }}-\omega_{\lambda^{\prime \prime }}\right)}{\omega_{\lambda }\omega_{\lambda^{\prime }}\omega_{\lambda^{\prime \prime }}}{\left|V_{\lambda \lambda^{\prime }\lambda^{\prime \prime }}^{\pm }\right|}^{2}.$$

Subscript *λ* represents the phonon mode. Both emission and absorption processes of three-phonon scattering are included in Equation (3). Similarly, the $\frac{1}{\tau_{\lambda }^{4ph}}$ is obtained by summing four-phonon transition probabilities $\Gamma_{\lambda \lambda^{\prime }\lambda^{\prime \prime }\lambda^{\prime \prime \prime }}^{\pm \pm }$, as expressed in the following equation [35].


(4) $$\Gamma_{\lambda \lambda^{\prime }\lambda^{\prime \prime }\lambda^{\prime \prime \prime }}^{\pm \pm }=\frac{\hbar^{2}\pi }{8N}\left\{\begin{array}{l} (1+f_{\lambda^{\prime }})(1+f_{\lambda^{\prime \prime }})f_{\lambda^{\prime \prime \prime }}\\ (1+f_{\lambda^{\prime }})f_{\lambda^{\prime \prime }}f_{\lambda^{\prime \prime \prime }}\\ f_{\lambda^{\prime }}f_{\lambda^{\prime \prime }}f_{\lambda^{\prime \prime \prime }} \end{array}\right\}\frac{\delta \left(\omega_{\lambda }\pm \omega_{\lambda^{\prime }}\pm \omega_{\lambda^{\prime \prime }}-\omega_{\lambda^{\prime \prime \prime }}\right)}{f_{\lambda }\omega_{\lambda }\omega_{\lambda^{\prime }}\omega_{\lambda^{\prime \prime }}\omega_{\lambda^{\prime \prime \prime }}}{\left|V_{\lambda \lambda^{\prime }\lambda^{\prime \prime }\lambda^{\prime \prime \prime }}^{\pm \pm }\right|}^{2}.$$

Equation (4) describes the splitting, redistribution and recombination channels involved in four-phonon scattering processes.

## 3. Results and Discussion

**Figure 1:**
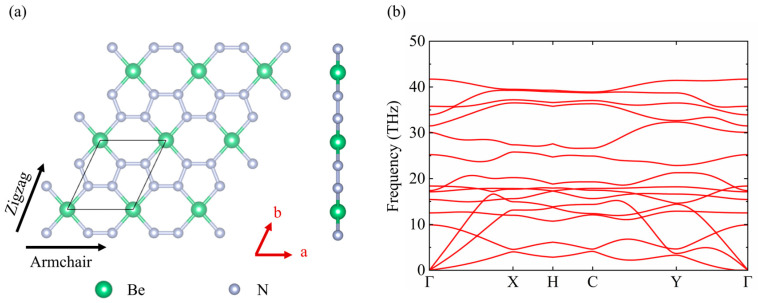
(**a**) Top and side views and (**b**) phonon dispersion of BeN_4_ monolayer. The black parallelogram represents the unitcell.

As shown in Figure 1a, the BeN_4_ monolayer exhibits a honeycomb-like structure with the space group of P2/m. In the BeN_4_ monolayer, each Be atom is bonded to four N atoms, while each N atom is connected to one Be atom and two N atoms. The thickness of the BeN_4_ monolayer is 3.45 Å, which is the sum of the van der Waals radii of Be (1.9 Å) and N (1.55 Å) [38]. The optimized lattice parameters are *a* = 3.66 Å and *b* = 4.27 Å, with an in-plane lattice angle of 64.62°. These values are in agreement with previous DFT results [39] (*a* = 3.66 Å, *b* = 4.27 Å, and 64.64°), and are similar with original experimental parameters (*a* = 3.28 Å, *b* = 4.21 Å, and 68.14°) [19]. Figure 1b illustrates the phonon dispersion of the BeN_4_ monolayer. No imaginary phonon frequencies are observed, confirming the dynamic stability of the structure. A total of 15 phonon branches are identified, consisting of 3 acoustic modes and 12 optical modes. The flexural acoustic (ZA) phonon branch exhibits parabolic near the Γ point, whereas the longitudinal acoustic (LA) and the transverse acoustic (TA) phonon branches exhibit linear dispersions. Such characteristics are commonly observed in 2D materials [24,40].

**Figure 2:**
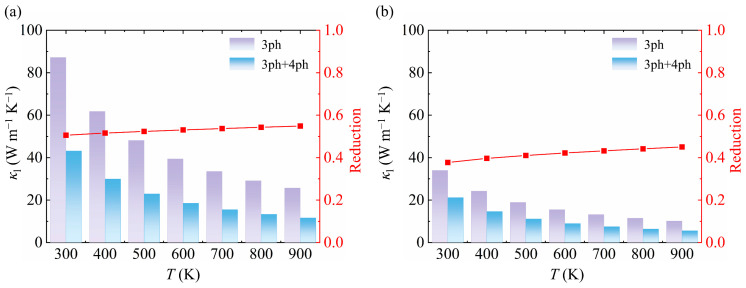
*κ*_3ph_ and *κ*_3ph+4ph_ values in the (**a**) armchair and (**b**) zigzag directions. The red squares indicate the reduction in *κ*_l_ due to 4 ph scattering.

The *κ*_l_ of the BeN_4_ monolayer along the armchair and zigzag directions is calculated over the temperature range of 300~900 K, as presented in Figure 2. We first consider the 3 ph scattering process, denoted as *κ*_3ph_, and subsequently include the 4 ph scattering process, denoted as *κ*_3ph+4ph_. The results clearly demonstrate significant anisotropy in the thermal properties, with the armchair direction exhibiting stronger thermal transport capability than the zigzag direction. The anisotropy of BeN_4_ is consistent with previous studies: the anisotropy is mainly associated with direction-dependent phonon dispersions and group velocities, which lead to more efficient heat transport along the armchair direction [24]. At 300 K, the *κ*_3ph_ values along the armchair and zigzag directions are 87.2 and 34.0 W m^−1^ K^−1^, respectively. As the temperature increases, the *κ*_3ph_ decreases substantially in both directions, reaching 25.7 and 10.2 W m^−1^ K^−1^ at 900 K, respectively. After including 4 ph scattering, the *κ*_l_ decreases significantly. At 300 K, the *κ*_3ph+4ph_ along the armchair direction is reduced to 43.1 W m^−1^ K^−1^, corresponding to a reduction of 50.6%. Along the zigzag direction, the corresponding values are 21.2 W m^−1^ K^−1^ and 37.7%. These results indicate that 4 ph scattering makes a substantial contribution to the thermal transport of the BeN_4_ monolayer and cannot be neglected in either direction. Furthermore, the reduction in *κ*_l_ becomes more pronounced with increasing temperature, reaching 54.9% along the armchair direction and 45.0% along the zigzag direction at 900 K.

**Figure 3:**
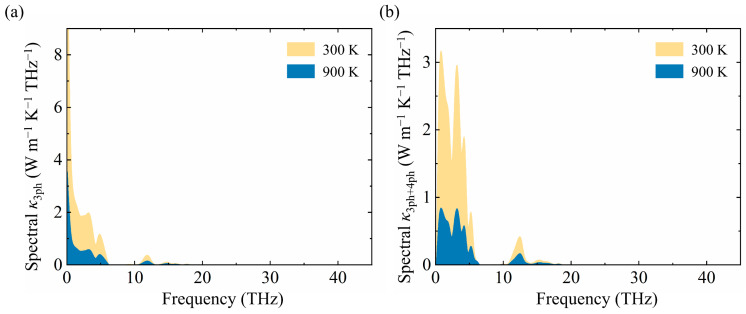
(**a**) Spectral *κ*_3ph_ and (**b**) spectral *κ*_3ph+4ph_ as a function of frequency for BeN_4_ monolayer at 300 K and 900 K.

Figure 3 presents the spectral contributions to *κ*_3ph_ and *κ*_3ph+4ph_ at 300 and 900 K. Acoustic phonons contribute much more significantly to thermal transport than optical phonons, particularly in the frequency range of 0~6 THz, which dominates both *κ*_3ph_ and *κ*_3ph+4ph_. Compared with the case considering only 3 ph scattering, the relative contribution of optical phonon modes to the total thermal conductivity increases slightly after including 4 ph scattering. Another notable feature is that the contribution of low-frequency acoustic phonon modes within 0~1 THz significantly decreases with the addition of 4 ph scattering, while more phonon modes with frequencies in the range of 1~5 THz participate in thermal transport. In particular, the ZA branch provides a dominant contribution to thermal transport, as will be further evidenced by the phonon scattering analysis presented below. In addition, the temperature-induced reduction in thermal conductivity is mainly concentrated in the low-frequency region.

**Figure 4:**
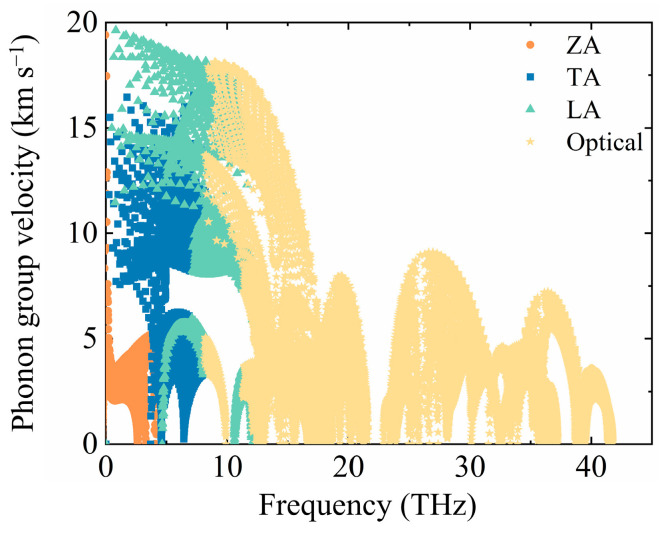
Phonon group velocities for BeN_4_ monolayer.

Figure 4 shows the phonon group velocity, which is determined by the slope of each phonon branch. Near 0 THz, the LA branch exhibits the highest group velocity. Overall, the acoustic phonon branches possess significantly higher phonon group velocities than the optical phonon branches. To quantify this difference, the averaged phonon group velocities for the acoustic and optical phonons are calculated to be 5.92 and 3.02 km s^−1^, respectively. The larger group velocities of the acoustic phonons explain why they dominate the lattice thermal transport in the BeN_4_ monolayer.

**Figure 5:**
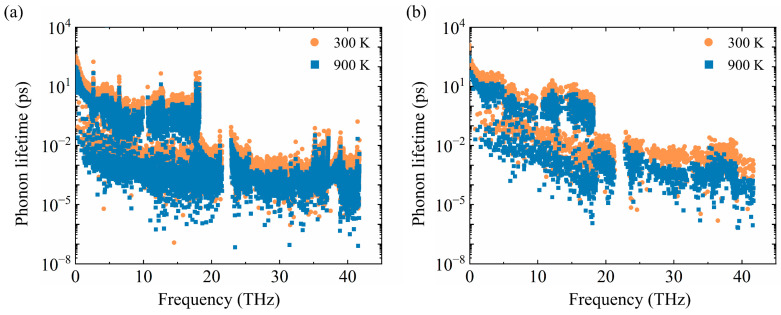
(**a**) The 3 ph phonon lifetime and (**b**) 3 ph + 4 ph phonon lifetime for the BeN_4_ monolayer at 300 K and 900 K.

**Figure 6:**
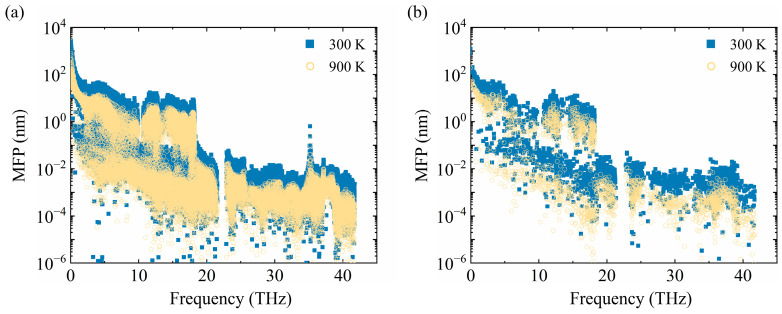
(**a**) The 3 ph MFP and (**b**) 3 ph + 4 ph MFP for the BeN_4_ monolayer at 300 K and 900 K.

In addition to phonon group velocity, phonon lifetime also plays a crucial role in determining the lattice thermal conductivity. As illustrated in Figure 5, the phonon lifetimes at 900 K are significantly reduced compared with those at 300 K for both the case considering only 3 ph scattering and that including the 3 ph + 4 ph scattering processes. To comprehensively evaluate the combined effects of phonon group velocity and phonon lifetime, the phonon mean free path (MFP) is further calculated. The MFP is defined as the product of phonon group velocity and phonon lifetime, and the results are presented in Figure 6. It can be observed that the MFPs at 300 K are considerably larger than those at 900 K, which is consistent with the much higher *κ*_l_ at low temperature. In particular, the MFPs of low-frequency acoustic phonons within 0~1 THz decrease significantly after including 4 ph scattering, in agreement with the spectral *κ*_l_ shown in Figure 3.

**Figure 7:**
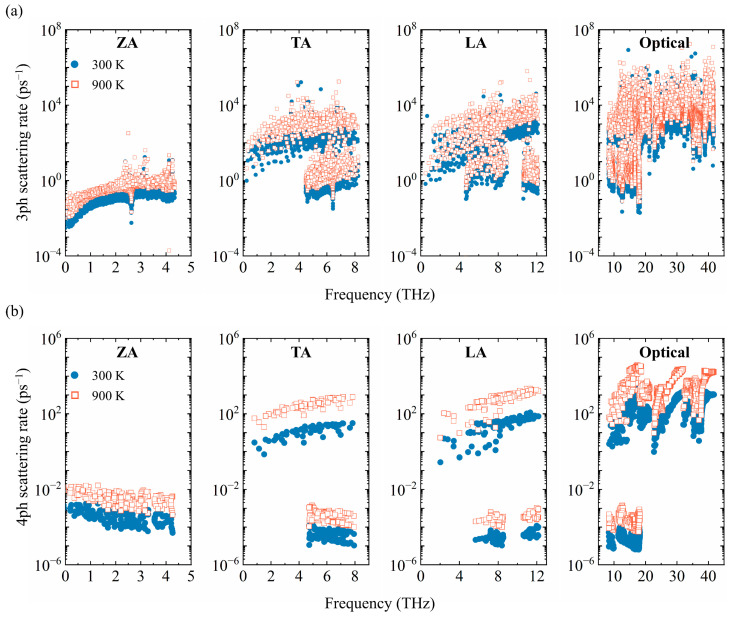
(**a**) The 3 ph scattering rate and (**b**) 4 ph scattering rate of different phonon modes for the BeN_4_ monolayer at 300 K and 900 K.

To investigate the microscopic scattering mechanisms, the 3 ph and the 4 ph scattering rates for different phonon modes of the BeN_4_ monolayer are analyzed in Figure 7. Overall, the 3 ph scattering rates are slightly larger than the corresponding 4 ph scattering rates. In addition, the optical phonons exhibit much larger scattering rates than the acoustic phonons, indicating their relatively minor contribution to lattice thermal transport. For the LA, TA, and optical phonon modes, both the 3 ph and 4 ph scattering rates generally increase with increasing phonon frequency. Specifically, the 4 ph scattering rates of the ZA phonon branch decrease with increasing frequency. This unusual behavior of ZA 4 ph scattering explains the relatively small contribution of low-frequency phonons within 0~5 THz to *κ*_3ph+4ph_. Similarly, the relatively low 4 ph scattering rates of optical phonons within the 10~20 THz frequency range are associated with the increased participation of optical modes in thermal transport after incorporating 4 ph scattering. Furthermore, both the 3 ph and 4 ph scattering rates exhibit strong temperature dependence, with substantially enhanced scattering at elevated temperatures. This behavior leads to a significant reduction of lattice thermal conductivity at high temperatures. From 300 to 900 K, *κ*_3ph_ decreases by approximately 70%, while *κ*_3ph+4ph_ decreases by 73%. The slightly larger reduction in *κ*_3ph+4ph_ can be attributed to the stronger temperature sensitivity of 4 ph scattering: the 4 ph scattering rates at 900 K are nearly one order of magnitude larger than those at 300 K.

**Figure 8:**
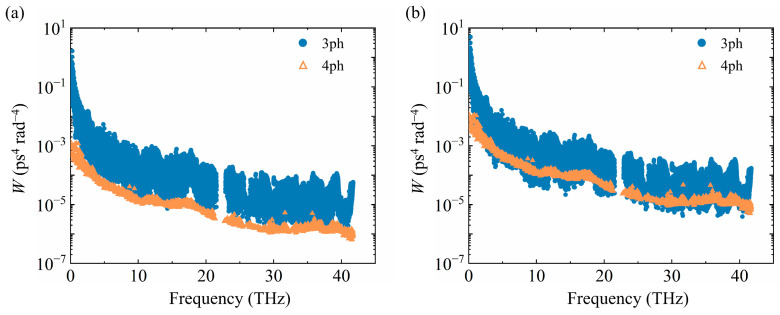
3 ph *W* and 4 ph *W* for BeN_4_ monolayer at (**a**) 300 K, and (**b**) 900 K, respectively.

To light the microscopic origin of the 3 ph and 4 ph scattering rates, the 3 ph and 4 ph weighted phase space (*W*) at 300 and 900 K are analyzed in Figure 8. The *W* is a direct measure of the transition probabilities of the phonon scattering processes [41]. The 3 ph *W* of acoustic modes in the 0~1 THz frequency range is extremely large, indicating that these phonons have a higher probability of scattering, which reflects their important role in thermal transport. When the temperature increases, the *W* of 3 ph and 4 ph processes are all enhanced. In addition, the 3 ph *W* is totally higher than 4 ph *W* at 300 K, whereas at 900 K, some phonon modes’ 3 ph *W* are smaller than their 4 ph *W*, indicating that the 4 ph process becomes more important at high temperature.

**Figure 9:**
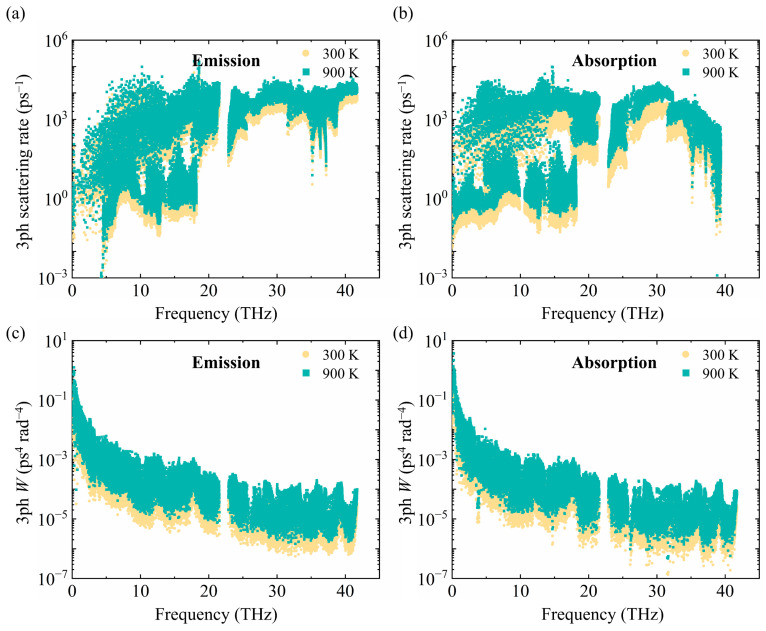
(**a**,**b**) 3 ph scattering rates and (**c**,**d**) 3 ph *W* for all channels at 300 and 900 K.

To gain deeper insight into the intrinsic mechanisms of 3 ph and 4 ph scattering, the absorption and emission processes, as well as their corresponding *W*, are systematically investigated. For 3 ph scattering, the absorption and emission processes can be expressed as *λ* + *λ*_1_ → *λ*_2_ and *λ* → *λ*_1_ + *λ*_2_, respectively. The *λ* denotes the phonon mode. As shown in Figure 9a,b, the scattering rates of the absorption processes decrease significantly in the high-frequency region. In contrast, the emission processes exhibit relatively low scattering rates in the low-frequency region. These differences are due to energy conservation restrictions. In Figure 9c,d, both the emission and absorption scattering probabilities decrease notably with increasing frequency. The low-frequency acoustic phonons show high probabilities for both emission and absorption processes. As the temperature increases from 300 to 900 K, the *W* increases, thereby strengthening phonon scattering and contributing to the reduction of *κ*_l_ at high temperature.

**Figure 10:**
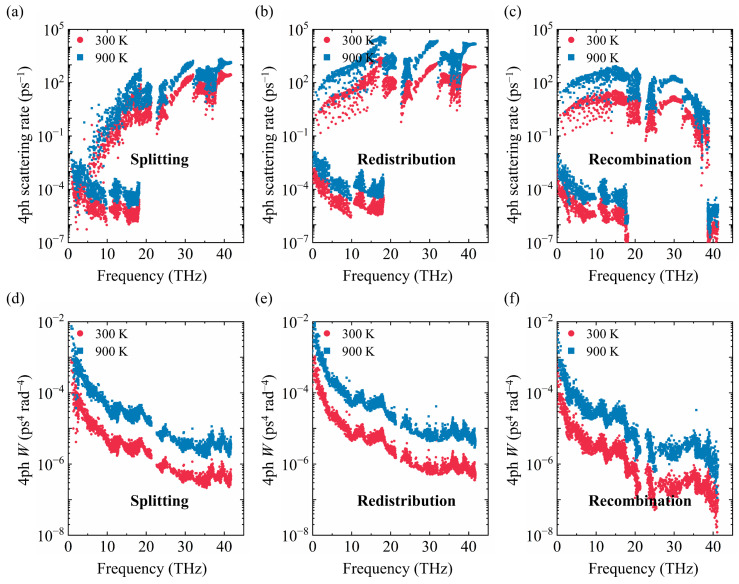
(**a**–**c**) 4 ph scattering rates and (**d**–**f**) 4 ph *W* for all channels at 300 and 900 K.

For 4 ph scattering, the splitting, redistribution, and recombination processes can be represented as *λ* → *λ*_1_ + *λ*_2_ + *λ*_3_, *λ* + *λ*_1_ → *λ*_2_ + *λ*_3_, and *λ* + *λ*_1_ + *λ*_2_ → *λ*_3_, respectively. The scattering rates and *W* of these processes are presented in Figure 10. Among these processes, the redistribution channel exhibits significantly larger scattering rates than the splitting and recombination channels over the entire frequency range. Moreover, the recombination scattering rates decrease substantially in the high-frequency range, whereas the splitting scattering rates are strongly suppressed in the low-frequency range and vanish near 0 THz. These characteristics also originate from the constraints imposed by energy conservation: recombination processes are difficult to occur at high frequencies, while splitting processes are restricted at low frequencies. In contrast, redistribution processes can satisfy energy conservation more easily across a broad frequency range, making them the dominant mechanism in 4 ph scattering. This conclusion is further supported by the *W* analysis. Although the *W* of all three 4 ph processes decreases with increasing frequency, the redistribution process consistently possesses a larger *W* than the other two processes. In addition, increasing temperature enlarges both the scattering rates and *W* of all scattering channels, thereby further suppressing thermal transport.

To further understand the anharmonicity of the BeN_4_ monolayer, the Grüneisen parameter [42,43] (*γ*) is calculated. The *γ* is a dimensionless quantity describing the strength of anharmonic interactions and can be determined from the dependence of phonon frequency *ω* and volume *V*, as expressed in $\gamma =-\frac{V}{\omega }\frac{\partial \omega }{\partial V}$. The calculated *γ* values at 300 and 900 K are 4.5 and 5.3, respectively. The larger *γ* at high temperature indicates enhanced anharmonicity, which strengthens phonon scattering and consequently leads to lower *κ*_l_.

## 4. Conclusions

In conclusion, we comprehensively explore the effects of 4 ph scattering and temperature on the *κ*_l_ of the BeN_4_ monolayer through the first-principles calculation. After incorporating 4 ph scattering, the *κ*_l_ at 300 K decreases by 50.6% and 37.7% along the armchair and zigzag directions, respectively. Moreover, stronger 4 ph scattering at elevated temperatures leads to a more pronounced reduction in *κ*_l_. Detailed phonon transport analysis reveals that the ZA phonon branch dominates the thermal transport of the BeN_4_ monolayer. Owing to energy conservation limitations, the recombination scattering rates become significantly weakened at high frequencies, whereas the splitting scattering rates are largely suppressed in the low-frequency region. In contrast, the redistribution channel can satisfy energy conservation conditions over a broader frequency range, making it the dominant contribution to the 4 ph scattering process. In addition, the reduced phonon mean free path, enhanced 3 ph/4 ph scattering rates, enlarged 3 ph/4 ph weighted phase space, and increased Grüneisen parameter at high temperature collectively contribute to the lower *κ*_l_. This work deeply reveals the mechanisms of 4 ph scattering in the 2D BeN_4_ monolayer, which can provide a theoretical basis for future applications in nanoelectronic and thermal management.

## Data Availability

The original contributions presented in this study are included in the article. Further inquiries can be directed to the corresponding author.
